# Supplementary material for: The impact of outdoor walking interventions on frailty among older adults with mobility limitations: Findings from the Getting Older Adults Outdoors (GO-OUT) study
Source: PLoS One. 2025 Sep 12;20(9):e0323923. doi: 10.1371/journal.pone.0323923 (PMC12431197; doi:10.1371/journal.pone.0323923)
Supplement: S1 Fig — (PDF) [file pone.0323923.s001.pdf]

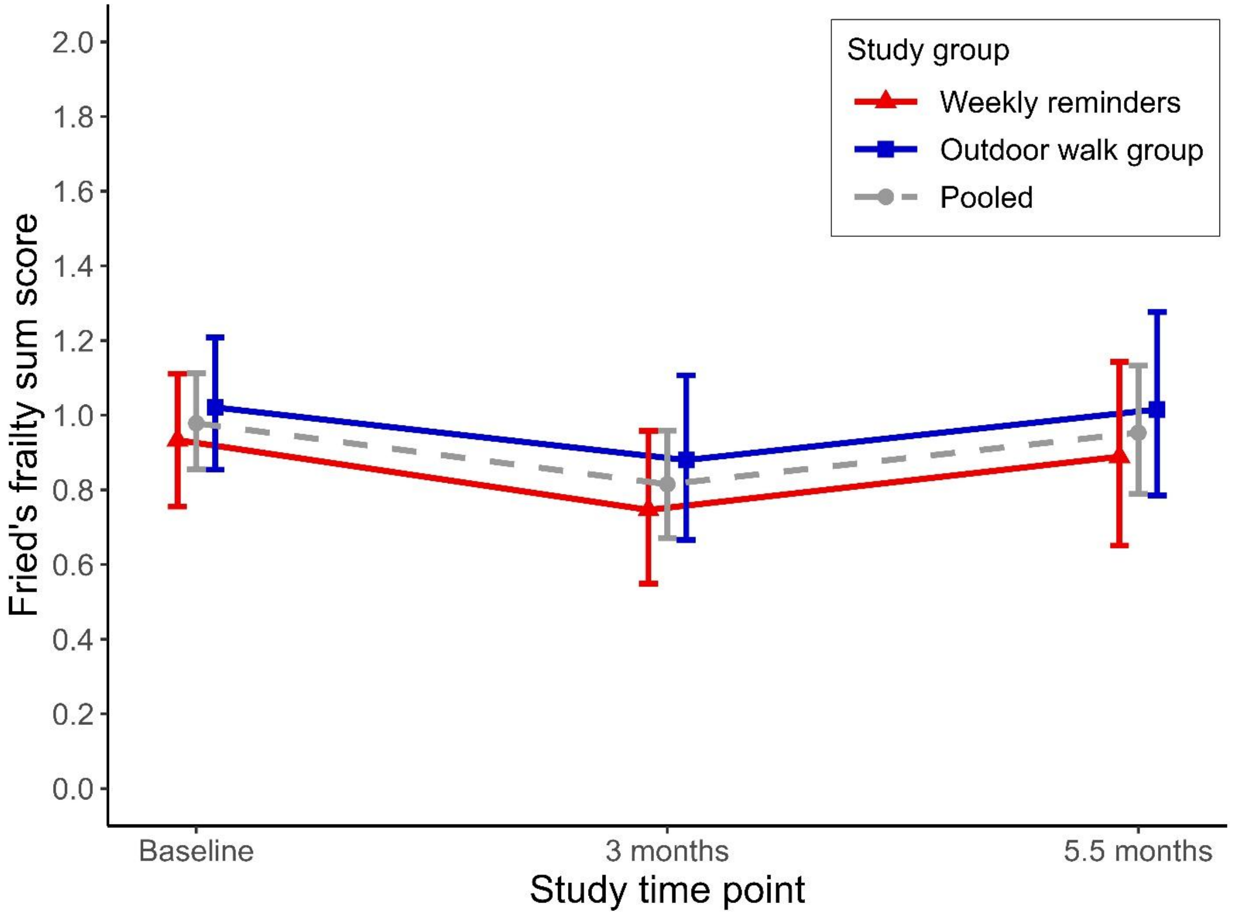

S1 Fig. Frailty sum score within and after pooling intervention groups at each evaluation time point
