# Supplementary material for: The impact of outdoor walking interventions on frailty among older adults with mobility limitations: Findings from the Getting Older Adults Outdoors (GO-OUT) study
Source: PLoS One. 2025 Sep 12;20(9):e0323923. doi: 10.1371/journal.pone.0323923 (PMC12431197; doi:10.1371/journal.pone.0323923)
Supplement: S1 Table — (PDF) [file pone.0323923.s003.pdf]

**S1 Table 1**. Summary of missing frailty scores at each study time point

| | Number of participants with missing frailty score* | | |
|---|---|---|---|
| Study time point | Pooled | Outdoor walk group | Weekly reminders |
| Baseline | 4 (2%) | 2 (2%) | 2 (2%) |
| 3 months | 6 (4%) | 3 (4%) | 3 (4%) |
| 5.5 months | 8 (6%) | 5 (7%) | 3 (5%) |
| 12 months | **88 (69%)** | **43 (67%)** | **45 (70%)** |

*Note*: *Due to COVID-19 precautions, we were unable to collect performance-based outcome measures for frailty assessment, including grip strength and walking speed, from participants in Cohort 2 at the 12-month time point. Hence, we focused on data collected at baseline, 3 months, and 5.5 months for the present study.
