# Supplementary material for: The impact of outdoor walking interventions on frailty among older adults with mobility limitations: Findings from the Getting Older Adults Outdoors (GO-OUT) study
Source: PLoS One. 2025 Sep 12;20(9):e0323923. doi: 10.1371/journal.pone.0323923 (PMC12431197; doi:10.1371/journal.pone.0323923)
Supplement: S2 Table — (PDF) [file pone.0323923.s004.pdf]

S2 Table. Baseline characteristics of participants who remained in the GO-OUT study and those who withdrew the subsequent evaluation time points (3 or 5.5 months)

Participant characteristics (units or scoring)	Completers (n = 133)	Withdrawals (n = 57)
	Mean ± SD / n (%)	
Intervention group		
Outdoor walk group	69 (52)	29 (51)
Weekly reminders group	64 (48)	28 (49)
Study site		
Site 1	32 (24)	19 (33)
Site 2	42 (32)	11 (19)
Site 3	34 (26)	16 (28)
Site 4	25 (19)	11 (19)
Participant type		
Individual	107 (80)	47 (82)
Dyad	26 (20)	10 (18)
Cohort		
2018-19	52 (39)	13 (23)
2019-20	81 (61)	44 (77)
Age (years)	74.53 ± 6.73	74.30 ± 8.03
Sex		
Male	35 (26)	16 (28)
Female	98 (74)	41 (72)
Body mass index (kg/m^2)	29.09 ± 5.91	30.40 ± 7.10
Educational attainment		
Secondary or lower	28 (21)	11 (19)
Some or completed college	48 (36)	20 (35)
Bachelor’s degree or higher	57 (43)	26 (46)
Uses a walking aid	31 (23)	17 (30)
Charlson comorbidity index (0–39)	1.79 ± 1.69	2.48 ± 2.29
6-minute walk test (m)	357.83 ± 89.95	360.01 ± 94.79
10-meter walk test at comfortable pace (m/s)	1.08 ± 0.22	1.06 ± 0.26
10-meter walk test at fast pace (m/s)	1.43 ± 0.30	1.37 ± 0.35
Mini-BESTest (0–28) †	20.89 ± 4.66	19.32 ± 6.21
30-second sit-to-stand (count)	8.62 ± 3.84	7.67 ± 4.49
ASCQ (0–10) ‡	8.03 ± 1.48	7.60 ± 1.87
RAND-36 emotional well-being (0–100)	77.83 ± 14.63	71.14 ± 16.51
Frailty score (0–5)	0.89 ± 0.86	1.20 ± 0.98
Frailty phenotype		
Non-frail (0)	50 (38)	13 (24)
Pre-frail (1–2)	75 (57)	35 (65)
Frail (≥3)	7 (5)	6 (11)

Note: SD = standard deviation; m = meters; m/s = meters/second. †Mini-BESTest = Mini Balance Evaluation System test, with higher scores indicating better overall balance function; ‡ ASCQ = ambulatory self-confidence questionnaire, with higher scores indicating greater confidence with walking ability.
