# Supplementary material for: The impact of outdoor walking interventions on frailty among older adults with mobility limitations: Findings from the Getting Older Adults Outdoors (GO-OUT) study
Source: PLoS One. 2025 Sep 12;20(9):e0323923. doi: 10.1371/journal.pone.0323923 (PMC12431197; doi:10.1371/journal.pone.0323923)
Supplement: S3 Table — (PDF) [file pone.0323923.s005.pdf]

S3 Table. Presence of the five frailty indicators by intervention group and time point

	Pooled			Outdoor walk group			Weekly reminders		
	Baseline (n=190)	3 months (n=152)	5.5 months (n=136)	Baseline (n=98)	3 months (n=78)	5.5 months (n=70)	Baseline (n=92)	3 months (n=74)	5.5 months (n=66)
Frailty indicator	Number (%)			Number (%)			Number (%)		
Weakness	77 (42)	52 (35)	48 (38)	45 (47)	32 (42)	29 (45)	32 (36)	20 (28)	19 (30)
Exhaustion	49 (26)	37 (25)	33 (25)	26 (27)	18 (24)	20 (29)	23 (25)	19 (26)	13 (20)
Low activity	23 (13)	14 (10)	23 (19)	10 (11)	7 (9)	10 (16)	13 (15)	7 (10)	13 (22)
Weight loss	22 (12)	15 (10)	15 (11)	13 (13)	9 (12)	7 (10)	9 (10)	6 (8)	8 (12)
Slowness	12 (6)	3 (2)	9 (7)	5 (5)	0	3 (7)	7 (8)	3 (4)	6 (10)
