# Supplementary material for: The impact of outdoor walking interventions on frailty among older adults with mobility limitations: Findings from the Getting Older Adults Outdoors (GO-OUT) study
Source: PLoS One. 2025 Sep 12;20(9):e0323923. doi: 10.1371/journal.pone.0323923 (PMC12431197; doi:10.1371/journal.pone.0323923)
Supplement: S4 Table — (PDF) [file pone.0323923.s006.pdf]

S4 Table. Results of mixed-effects logistic regression models of change in frailty over time in the pooled sample of GO-OUT participants (sensitivity analysis)

	Outcome 3 Being pre-frail or frail vs. non-frail			
	Model 1		Model 2	
Variables	OR [95% CI]	*p*	OR [95% CI]	*p*
Time				
Baseline	*Reference*		*Reference*	
3 months	0.38 [0.19, 0.77]	.007	0.38 [0.19, 0.76]	.006
5.5 months	0.78 [0.38, 1.58]	.484	0.75 [0.37, 1.52]	.423
Intervention				
Weekly reminders	*Reference*		*Reference*	
Outdoor walk group	1.88 [0.61, 5.78]	.273	1.80 [0.67, 4.87]	.244
Age†	—		1.09 [1.00, 1.18]	.045
Sex	—			
Male			*Reference*	
Female			0.16 [0.05, 0.54]	.003
Type	—			
Individual			*Reference*	
Dyad			0.60 [0.17, 2.15]	.431
Cohort	—			
2018-19			*Reference*	
2019-20			0.57 [0.19, 1.66]	.300
Study site	—			
Site 1			*Reference*	
Site 2			5.58 [1.20, 25.97]	.029
Site 3			11.55 [2.48, 53.88]	.002
Site 4			1.53 [0.42, 5.57]	.514
Variance components				
Intercept (U_{0i})	9.38		6.28	

Note: OR = Odds ratio. CIs= Confidence intervals. Model 2 controlled for participants age, sex, and study clustering variables including study site, participant type, and cohort. † Age was centered at 63 years, representing the minimum age among the study participants.
